# Acceptance of an Internet-Based Team Development Tool Aimed at Improving Work-Related Well-being in Nurses: Cross-sectional Study

**DOI:** 10.2196/36702

**Published:** 2022-04-22

**Authors:** Sylvia Broetje, Georg F Bauer, Gregor J Jenny

**Affiliations:** 1 Center of Salutogenesis Epidemiology, Biostatistics and Prevention Institute University of Zurich Zurich Switzerland

**Keywords:** digital intervention, eHealth, nurses, online intervention, organizational health, technology acceptance, UTAUT, workplace health promotion, mHealth

## Abstract

**Background:**

Workplace health interventions can produce beneficial health- and business-related outcomes. However, such interventions have traditionally focused on lifestyle behaviors of individuals, mostly not considering the role of working conditions. The wecoach intervention is an internet-based tool that combines both a digital and a participatory team development approach aimed at addressing critical job demands and resources as key aspects of health-promoting working conditions. Nursing staff are particularly affected by challenging working conditions and could potentially benefit greatly. Understanding the acceptance of novel workplace health promotion approaches is a critical precursor to their successful implementation and use.

**Objective:**

This study aims to examine the factors influencing the acceptance of a digitally supported team development tool among nurse managers.

**Methods:**

A sample of 32 nurse managers from 3 German-speaking countries tested wecoach and completed our online questionnaire. Hypotheses were based on the unified theory of acceptance and use of technology (UTAUT) and the organizational health development (OHD) model and were tested using multiple regression analyses.

**Results:**

Our analyses found that merely capacities on the team level (CapTeam) significantly contributed to the acceptance of wecoach, although only after the other variables were excluded in the stepwise multiple regression analysis. The UTAUT predictors were unable to add significant variance explanation beyond that, and their inclusion masked the contribution of CapTeam.

**Conclusions:**

For the acceptance of a digitally supported participatory tool, the fit with the team, its culture, and its motivation are of critical importance, while aspects proposed by traditional acceptance models, such as the UTAUT, may not be applicable.

## Introduction

### Workplace Health Interventions

Workplace health programs can produce beneficial health- and business-related outcomes [1-3]. Such interventions have traditionally focused on targeting lifestyle behaviors of individuals, supporting them, for example, in quitting smoking, increasing their physical activity, or managing their stress levels. The role played by working conditions, however, has received little attention in the development of workplace health promotion programs [4]. A model well suited to studying and assessing the well-being-enhancing and well-being-diminishing aspects of work is the Job Demands-Resources model [5]. It proposes a dual pathway. Job resources, such as autonomy and social support, are linked to motivational outcomes, such as work engagement, while high levels of job demands, such as work interruptions or role conflict, are linked to strain and health impairment. Its assumptions have found widespread support in empirical research [6]. Data collected during a large-scale stress management intervention conducted in Switzerland showed that a favorable ratio of job resources to job demands is associated with lower exhaustion and absenteeism as well as higher engagement and productivity of staff [7]. Reviews also indicate that at least some organization-level interventions aimed at improving working conditions can positively affect outcomes such as mental health, physical health, absenteeism, or staff turnover [8,9].

Teams are optimal units for workplace health promotion [10]. Leaders not only play an important role in the implementation of interventions [11], but teams and leaders are also the level on which many job demands and resources are created. Teams can apply the expertise about their workplace to develop measures that are tailored to their own situation, and the participation of the team in this process enhances ownership of the intervention and facilitates learning and communication.

At the same time, the ongoing megatrend of digitization has led to an increase in the delivery of interventions in digital format. The most common forms are health apps, wearables, and health portals [12]. Such approaches have been shown to improve mental health in general population samples [13] as well as in employees [14]. However, no digital interventions have, to the best of our knowledge, aimed to improve health and well-being in employees via the improvement of working conditions.

This presents a highly innovative form of workplace health intervention. Previous intervention research has focused on the effectiveness of workplace health interventions, with aspects of acceptance and implementation receiving little attention. This, however, is changing. Attention is now directed towards *realist* evaluations of interventions, acknowledging the entire intervention process [15] as well as aspects preceding the use of an intervention, as emphasized by the *adoption* dimension of the Reach Effectiveness Adoption Implementation Maintenance (RE-AIM) framework [16,17]. If digital workplace health interventions are to fulfill their potential, they not only must be effective and well implemented, supporting their internal and external validity, but also need to be accepted by potential users. According to Rogers [18], the adoption of innovations is a 5-step process, leading from (1) knowledge about the product to (2) persuasion of the product, (3) decision to adopt—or reject—the product to its (4) implementation and (5) confirmation that one has made the right decision. During the stage of persuasion, an opinion about the product is formed, which is influenced by different characteristics of the product. These characteristics stem from attributes of the product itself, as well as from relevant outside factors, such as current needs or compatibility with other products. In accordance with this model, we view acceptance as the phase of formation of attitudes and usage intentions that precedes the adoption of a product.

Health care is one of the industrial sectors with the highest levels of health risks. The sixth European working conditions survey [19] names the health sector as the one with the highest work intensity index, measuring aspects such as quantitative demands, time pressure, interruptions, and emotional demands. It also reports that health sector workers have a substantially above average experience of adverse social behavior at work. Approximately one-third of nurses in Europe and the United States feel burned out [20], and 33% of nurses report wanting to leave their current employer within the next year due to job dissatisfaction, with 9% intending to leave the profession altogether [21]. Poor working conditions are at the root of this situation, with low pay, limited educational and career opportunities, unsafe workplaces, and lack of resources, contributing to nurse turnover and health impairment [22]. Although these challenges also need to be addressed on the societal and political levels, interventions on the level of the individual health care organization and even on the unit or team level can make a contribution to the improvement of working conditions [23].

### The Wecoach Approach

**Figure 1:**
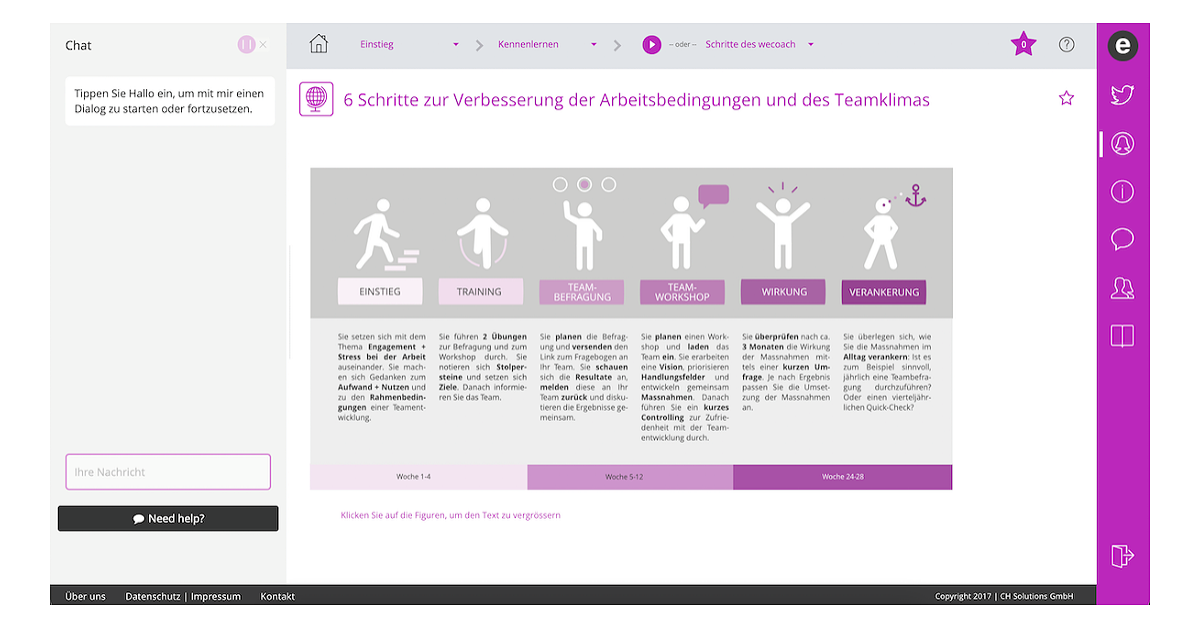
Screenshot of the wecoach main page.

**Figure 2:**
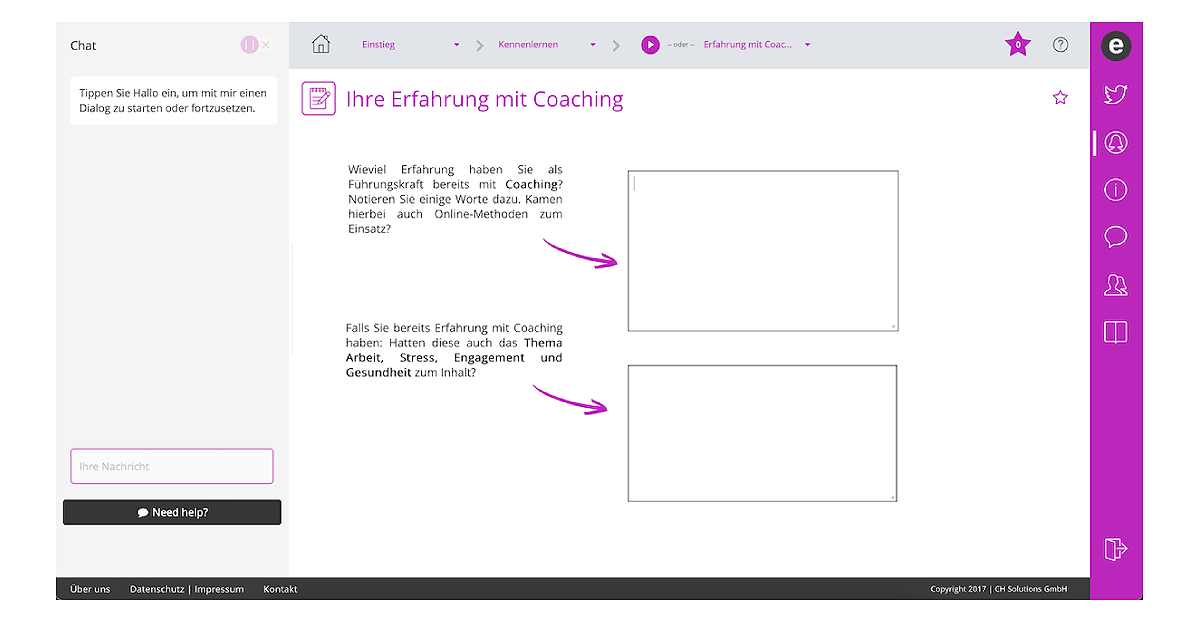
Screenshot of a wecoach interactive form.

In this study, we examine what affects the acceptance of wecoach, an internet-based tool that combines both a digital and a participatory team approach and guides team leaders through a health-oriented team development process. It is currently only available in German. When logging into wecoach, the team leader interacts with a chatbot, which advises the leader on which training session to complete next and presents training materials on work and health, self-assessments, and online tools to conduct team surveys and workshops, as well as self-evaluation of progress and effectiveness (see Figures 1 and 2). After the initial training sessions, the entire team is involved and team members complete a survey that assesses job demands and resources with validated scales, such as the Health and Safety Executive (HSE) Stress Management Standards [24], which have been found to be relevant across many industries. Afterward, a team workshop is conducted that builds on these results, developing measures to reduce demands or strengthen resources. The team workshop is moderated by the team leader, who has been provided with material on how to organize and conduct the workshop. Instructions on maintaining the effects of the intervention and a health-oriented team culture are also included. This sequence of sensitizing participants to the relationship between work and health, providing information and enhancing self-efficacy in team leaders, followed by assessments and team workshops, has been honed by our department in many on-site interventions, and these experiences built the foundation for the digitally supported wecoach intervention. The wecoach tool is based on a capacity-building approach, which the World Health Organization’s International Classification of Health Interventions describes as “providing resources or initiating strategies to increase the ability of an organisation or community to address health issues by creating new structures, approaches or values in relation to patterns of behaviour that may affect psychological health and wellbeing” [10,25].

### Study Aim and Hypotheses

The aim of this study is to examine the factors determining the acceptance of wecoach among nurse managers in 3 German-speaking countries in order to make a contribution to the understanding on what factors can help promote the use of participatory, digitally supported workplace interventions that can help address working conditions in a challenging work environment such as health care. We based our hypotheses regarding its acceptance on 2 models: the unified theory of acceptance and use of technology (UTAUT) and the organizational health development (OHD) model to capture the complexity of wecoach, which is simultaneously a technological innovation as well as an innovative participatory team approach.

#### Unified Theory of Acceptance and Use of Technology

UTAUT [26] is 1 of the most widely used models of technology acceptance. It examines the factors that explain the intention to use new technologies, especially in organizational contexts. It was developed empirically and integrates elements from 8 established models, including traditional psychological theories such as the theory of reasoned action [27] and social cognitive theory [28,29], as well as other technology-related models, such as the technology acceptance model [30].

UTAUT proposes 4 predictors [26]. Performance expectancy describes the degree to which an individual believes that using the system will help them attain gains in job performance. Effort expectancy refers to the degree of ease associated with the use of the system. Social influence is the degree to which an individual perceives that important others believe they should use the new system. Facilitating conditions refers to the degree to which an individual believes that an organizational and technical infrastructure exists to support the use of the system. The model proposes 2 outcome variables that indicate acceptance of a new technology: (1) behavioral intention, which is the expressed intention to use the technology in the near future, and (2) use behavior, which indicates how often people, within a certain period after the technology rollout, actually use the technology, typically tracked by company logs. Based on the original 8 theories from which UTAUT was synthesized, gender, age, voluntariness, and experience were also included into the original model as moderators.

UTAUT has been applied in different contexts, especially to study the acceptance of online banking [31], general information technology [32], e-government services [33], or e-learning tools [34]. Among health care professionals, it has been used predominantly to examine factors influencing the acceptance of electronic medical records [35-37].

A meta-analysis of 74 studies by Khechine et al [38] confirmed the strength and robustness of UTAUT and corroborated findings from a previous meta-analysis by Taiwo and Downe [39]. All 4 predictors have been found to significantly predict behavioral intention and use behavior, respectively, with regression weights ranging from 0.4 to 0.5 [38]. Performance expectancy was the strongest predictor of behavioral intention, indicating that users will be keen to use a new technology when they believe that it would improve their productivity, efficiency, and effectiveness. The moderators proposed in UTAUT have rarely been examined in empirical studies [38,40] and were not considered in the meta-analysis. Venkatesh et al [40] also later distanced themselves from the inclusion of moderators in the model and suggested a focus on the main effects for enhanced parsimony.

As our study is exploratory in nature and we want to focus on the initial impressions potential users have of wecoach, we included only behavioral intention as an indicator of acceptance in this study. We expect all 4 predictors to be relevant to the acceptance of wecoach as a new technology. Our first hypothesis (H1) thus states:

- H1a: Performance expectancy contributes to the intention to use wecoach.
- H1b: Effort expectancy contributes to the intention to use wecoach.
- H1c: Social influence contributes to the intention to use wecoach.
- H1d: Facilitating conditions contribute to the intention to use wecoach.

#### Organizational Health Development Model

The wecoach tool is a complex intervention approach that is not only a new technology but also an innovative participatory approach that affects different organizational levels. For this reason, we considered it necessary to include additional predictors in our study. Attitudes or beliefs relating to the affected organizational levels may serve as the gateway to considering using such a tool, even before considering aspects such as usefulness or user-friendliness.

We included 3 variables from the OHD model [41]. The OHD model describes capacities on the individual and organizational levels that are needed to implement and sustain health interventions. For example, on the individual level, a leader needs to be competent and motivated to conduct an intervention and perceive it as fitting to their leadership style and values. These aspects should also be true for the members of their team. On the organizational level, resources should be available for the intervention and the intervention should fit with the goals and culture of the organization. Accordingly, we hypothesized that capacities on the individual leader's level (CapSelf), the team level (CapTeam), and the organizational level (CapOrg) will influence the intention to use wecoach. Our second hypothesis (H2) thus states:

- H2a: CapSelf contributes to the intention to use wecoach.
- H2b: CapTeam contributes to the intention to use wecoach.
- H2c: CapOrg contributes to the intention to use wecoach.

**Figure 3:**
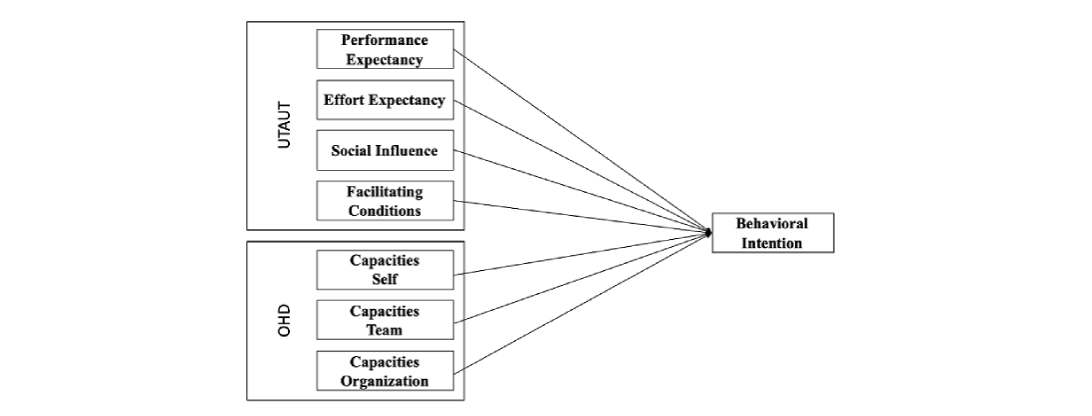
Proposed study model with predictors from UTAUT and the OHD model. OHD: organizational health development; UTAUT: unified theory of acceptance and use of technology.

Figure 3 illustrates our proposed study model with predictors from UTAUT and the OHD model.

## Methods

### Participants and Procedure

The participants in our study were nurse managers and nurse executives working in hospitals or nursing homes in Switzerland, Austria, and Germany. Nurses without leadership responsibilities were not included in the study. The rationale for this decision is that wecoach empowers team leaders to conduct a team development process, together with their staff, who do not directly interact with wecoach. Since nurse managers would be the primary users of wecoach, we were particularly interested in their acceptance of it.

Participants were identified by searching databases or publicly available lists of hospitals and nursing homes in all 3 countries. In some cases, an email address for the nursing director was directly available. In other cases, organization websites were listed, which were then searched for contact information of nursing directors, nurse managers. or other staff, such as human resource personnel, who might be in charge of team development or occupational health in nurses.

We contacted all the largest hospitals and nursing homes in all 3 countries. Additionally, using an online random generator, we also selected subsets of small and medium-size organizations in each canton or state. This varied slightly, based on the databases available for hospitals and nursing homes in each country; however, great efforts were undertaken to ensure that organizations of different sizes, from urban and rural areas and from all regions of each of the 3 countries, were included. The identified contacts were invited by email to participate in our study, and a flyer with further information was included. Participation in the study involved completing several modules of wecoach and then answering our online questionnaire (all in German; total time approximately 60-90 minutes). We sent out emails to 2269 recipients working in more than 500 organizations, deemed suitable for participating in or sharing the information about the study, such as nursing unit managers, nursing directors, or persons in charge of human resource development or occupational health and well-being. Persons interested in participating contacted the first author. To be included in the study, participants had to be working in a nursing leadership role with staff supervision responsibilities in either a hospital or a nursing home. Participants were asked to complete 4 modules of wecoach. The first module acquainted them with the technical interface, such as the chatbot and interactive forms. It also introduced them to general information about work and stress and asked them about their current level of confidence in undergoing health-oriented team development. The second module deepened the understanding of work, stress, and engagement; introduced users to the Job Demands-Resources model [5]; and provided an introduction to the team survey. In the third module, users learned how the team survey works and analyzed their own work situation. In the fourth module, they learned about the team workshop approach and practiced developing measures for improving 1 of their own job resources and job demands. These 4 modules represent only a selection of the full wecoach tool and were chosen to provide participants with a good overview of the team development approach and the technology of wecoach, while not requiring too much time. However, participants were free to move around wecoach and go over different modules as they pleased.

As an incentive, participants retained access to their fully active wecoach account, which allowed them to conduct an entire team development process free of charge. In total, 105 persons registered to participate in the study; however, many did not complete the wecoach modules or the questionnaire. The emails we received indicated that this was mainly due to time constraints. To encourage participation, we later provided an incentive of a raffle of 5 gift certificates for an online store worth €50 each (approximately US $54).

### Ethical Considerations

No ethical review of the study was necessary under federal, state, university, or departmental rules. The study was conducted under strict observation of ethical and professional guidelines.

### Measures

We assessed the variables of UTAUT by modifying the items used by Venkatesh et al [26]. We attempted to maintain the meaning of the original items, while adjusting them slightly for the purposes of our study. For example, we rephrased the statement *Using the system enables me to accomplish my tasks more quickly* to *I think that wecoach can enable me to more efficiently conduct team development*. The capacities for the team development approach were assessed with newly developed items based on the OHD model [41], with 3 items assessing each dimension. All UTAUT and OHD items are illustrated in Table 1. Participants responded to all of these on 7-point scales ranging from 1 (*strongly disagree*) to 7 (*strongly agree*). Additional data collected were demographics, work setting, leadership role, and voluntariness of testing wecoach.

**Table 1:** Items used to measure variables from UTAUT^a^ and the OHD^b^ model.

Variable	Items^c^
Behavioral intention	I intend to use wecoach within the next 6 months. I plan to use wecoach in the next 6 months. I mean to use wecoach in the next 6 months.
Performance expectancy	I find wecoach useful for conducting team development. I think that wecoach would make it easier for me to conduct team development. I think that wecoach can enable me to enhance the quality of team development. I think that wecoach can enable me to more efficiently conduct team development. I think that wecoach can convey the knowledge that I need to conduct team development.
Effort expectancy	I find that wecoach does what I want it to without problems. Operating wecoach is clear and easy to understand. Using wecoach does not require a lot of mental effort. I think that wecoach has intuitive user navigation. Learning to operate the system is easy.
Social influence	In general, I think that my organization would support the use of wecoach for team development. My fellow managers would support the use of wecoach for team development. My team would support the use of wecoach for team development. I think upper management would endorse the use of wecoach for team development. I would be more likely to use wecoach if my colleagues did so as well.
Facilitating conditions	I have the resources necessary to use wecoach. I have the technological know-how to be able to use wecoach. The wecoach tool is compatible with other systems I use. Assistance for using wecoach is available if I need it. Using wecoach fits with my way of working. Using wecoach fits with the human resource development strategy of our organization.
CapSelf^d^	I have the necessary competencies to do such team development. I am motivated to do such team development. This team development approach fits with my leadership style.
CapTeam^e^	Our team has the competences necessary to undertake such team development. Our team is motivated to do such team development. Such team development fits with our team culture.
CapOrg^f^	The necessary resources (time, finances) are available, so one can conduct such team development. Conducting such team development is in line with our organizational goals. Such team development fits well with our organizational culture.

^a^UTAUT: unified theory of acceptance and use of technology.

^b^OHD: organizational health development.

^c^Rated on a scale from 1 to 7.

^d^CapSelf: capacities on the individual leader's level.

^e^CapTeam: capacities on the team level.

^f^CapOrg: capacities on the organizational level.

### Analysis

All statistical analyses were performed using SPSS Statistics version 24. To test our hypotheses, we conducted multiple linear regression analyses with all variables that were significantly correlated with our outcome variable, behavioral intention. First, we used the enter method, followed by another analysis using the stepwise method.

## Results

### Sample Characteristics

In total, 36 participants reviewed wecoach and completed our questionnaire. Of these, 4 (11%) were removed from the analysis for the following reasons: not having a leadership role, not working in a hospital or long-term care setting, or not registering for the study. Our final sample consisted of 32 persons. Descriptive data on our sample are presented in Table 2.

**Table 2:** Sample characteristics (N=32).

Characteristic		Respondents	
Age (years), mean (SD)		40.56 (7.76)	
**Gender, n (%)**			
	Female	23 (71.9)	
	Male	9 (28.1)	
**Work setting, n (%)**			
	Hospital	28 (87.5)	
	Long-term care	2 (6.3)	
	Other	2 (6.3)	
**Work country, n (%)**			
	Switzerland	12 (37.5)	
	Austria	12 (37.5)	
	Germany	8 (25.0)	
**Leadership level, n (%)**			
	Upper	6 (18.8)	
	Middle	22 (68.8)	
	Lower	4 (12.5)	
**Voluntariness of testing wecoach, n (%)**			
	Own motivation	24 (75.0)	
	Were advised to	8 (25.0)	
Minutes spent in wecoach, mean (SD)		137.75 (103.21)	

### Preliminary Analyses

The internal reliabilities of our scales ranged from .72 to .92. All variables were examined for outliers based on 2.2 IQRs [42]. Two extreme low values were identified on the variable CapSelf and were winsorized by replacing them with the next lowest value that was not an outlier. Inspection of our outcome variable, behavioral intention, questioned its normal distribution, which was supported by a significant Shapiro-Wilk test (*P*=.02). Thus, we proceeded with our analyses using Spearman correlation analyses and the Kruskall-Wallis test for group comparisons.

We assessed group differences on the predictor and outcome variables based on sex, age, country, leadership level, and voluntariness of testing wecoach. No significant group differences were found in any of these. Note that no group comparisons were performed for work setting, since 28 (87.5%) of our final 32 participants worked in hospitals, while only 2 (6.25%) worked in long-term care and 2 (6.25%) in psychiatric acute care.

The variable of greatest interest to us was behavioral intention as an indicator of acceptance. Its mean level can be described as moderate. Of all assessed variables, it showed the highest degree of variability among participants. Table 3 displays the mean scores and SDs for behavioral intention and all predictors, their correlations, and internal reliabilities.

**Table 3:** Scores on UTAUT^a^ and OHD^b^ variables, correlations, and internal reliabilities^c^ (N=32).

Variable	Mean^d^ (SD)	Correlation (*P*)							
		Behavioral intention	Performance expectancy	Effort expectancy	Social influence	Facilitating conditions	CapSelf^e^	CapTeam^f^	CapOrg^g^
Behavioral intention	4.40 (1.94)	.92	N/A^h^	N/A	N/A	N/A	N/A	N/A	N/A
Performance expectancy	5.57 (0.99)	.49^i^	.92	N/A	N/A	N/A	N/A	N/A	N/A
Effort expectancy	5.57 (0.88)	.36^j^	.25	.81	N/A	N/A	N/A	N/A	N/A
Social influence	4.78 (1.03)	.53^i^	.50^i^	.48^i^	.79	N/A	N/A	N/A	N/A
Facilitating conditions	4.65 (0.98)	.52^i^	.59^k^	.53^i^	.78^k^	.72	N/A	N/A	N/A
CapSelf	5.81 (0.67)	.29	.39^j^	.50^i^	.37^j^	.37^j^	.81	N/A	N/A
CapTeam	4.80 (1.15)	.61^k^	.52^i^	.46^i^	.67^k^	.60^k^	.44^j^	.93	N/A
CapOrg	4.45 (1.36)	.34	.38^j^	.32	.65^k^	.57^i^	.40^j^	.63^k^	.87

^a^UTAUT: unified theory of acceptance and use of technology.

^b^OHD: organizational health development.

^c^Internal reliabilities are reported in the diagonal.

^d^Rated on a scale from 1 to 7.

^e^CapSelf: capacities on the individual leader's level.

^f^CapTeam: capacities on the team level.

^g^CapOrg: capacities on the organizational level.

^h^N/A: not applicable.

^i^*P*<.01.

^j^*P*<.05.

^k^*P*<.001.

### Findings

To test our hypotheses, the predictors that were significantly correlated with the outcome variable, behavioral intention, namely performance expectancy, effort expectancy, social influence, facilitating conditions, and CapTeam, were entered into a multiple regression model. The assumptions for linear regression were tested and all met, with the possible issue of multicollinearity between social influence and facilitating conditions, which correlated at .776 (*P*<.001). Examination of the collinearity statistics found the lowest tolerance for facilitating conditions at .27 (with a variance inflation factor of 3.66) and social influence at .28 (with a variance inflation factor of 3.58). Depending on the chosen cut-off, these values can still be considered tolerable.

We began by simultaneously including all 5 predictors using the enter method. This allowed us to examine the overall predictive power of the model as well as examine the respective β weights of the predictors in conjunction. The model explained 43.9% of the variance in behavioral intention (adjusted R^2^=.331). As Table 4 illustrates, none of the predictors reached significance. CapTeam was the strongest predictor, followed, in declining order, by performance expectancy, social influence, facilitating conditions, and, lastly, effort expectancy.

**Table 4:** Contributions to behavioral intention: multiple regression analysis using the enter method.

Variable	Unstandardized coefficient B	SE	Standardized β	*P* value	95% CI
(Constant)	–.3013	2.240	N/A^a^	.19	–7.617 to 1.591
Performance expectancy	.441	0.345	.226	.21	–.268 to 1.150
Effort expectancy	.015	0.450	.007	.97	–.817 to .847
Social influence	.405	0.525	.215	.45	–.674 to 1.484
Facilitating conditions	.129	0.554	.065	.82	–1.011 to 1.268
CapTeam^b^	.485	0.349	.288	.18	–.232 to 1.203

^a^N/A: not applicable.

^b^CapTeam: capacities on the team level.

Overlaps in explained variance may have caused the lack of any of the individual predictors reaching significance. To identify the most useful one(s), we also conducted stepwise multiple regression analysis. CapTeam was retained as the only predictor that uniquely contributed to behavioral intention with a standardized β of .582 (*P*<.001). This model explained 33.8% of the variance (adjusted R^2^=.316) in behavioral intention. All other variables were excluded from the model. These findings are illustrated in Table 5.

**Table 5:** Contributions to behavioral intention: stepwise multiple regression analysis.

Variable		Unstandardized coefficient B	SE	Standardized β	*P* value	95% CI
**Model 1**						
	(Constant)	–.313	1.235	N/A^a^	.80	–2.835 to 2.209
	CapTeam^b^	.981	0.250	.582	<.001	0.469-1.492
**Excluded variables**						
	Performance expectancy	.283	N/A	N/A	.094	N/A
	Effort expectancy	.093	N/A	N/A	.582	N/A
	Social influence	.324	N/A	N/A	.105	N/A
	Facilitating conditions	.285	N/A	N/A	.130	N/A

^a^N/A: not applicable.

^b^CapTeam: capacities on the team level.

A post hoc power analysis using G*Power (Faul, Erdfelder, Buchner, and Lang) [43] estimated the power of our regression analyses at .96, which is good and indicates that despite our limited sample size, our findings are interpretable.

Our findings were not able to confirm any of our hypotheses regarding the predictors of UTAUT (H1). None of the four predictors (ie, performance expectancy, effort expectancy, social influence, and facilitating conditions) significantly contributed to the acceptance of wecoach, indicated by behavioral intention. Of the 3 levels of capacities derived from the OHD model (H2), only CapTeam was found to be a significant predictor, although only after the other variables were excluded in the stepwise multiple regression analysis. Neither CapSelf nor CapOrg significantly contributed to behavioral intention. In summary, only H2b was partially confirmed.

## Discussion

### Principal Findings

The aim of our study was to examine factors that predict the acceptance of wecoach. In total, 32 nurse managers in Switzerland, Austria, and Germany tested several introductory modules of wecoach and completed our questionnaire, which assessed predictors from UTAUT [26] and the OHD model [41]. Although we hypothesized that all 7 would yield a significant influence on behavioral intention, our analyses found that merely CapTeam significantly contributed to the acceptance of wecoach, although only in the absence of the other predictors.

The level of behavioral intention to use wecoach was moderate, while both performance expectancy and effort expectancy were quite high. This suggests that although users perceived wecoach as rather useful, they also perceived it as requiring some effort.

Our findings raise the question of whether UTAUT was an appropriate model to determine acceptance in our study. We see findings similar to ours in a study by Apolinário-Hagen et al [44] in their examination of the acceptance of a stress management app. Their strongest predictor was attitudes about the use of health apps for stress management, and like in our study, no additional significant contribution by the UTAUT predictors was found. They concluded that attitudes “may be a more relevant initial precondition of acceptance than elaborated cognitive beliefs on usefulness or usability” [44]. Another, albeit smaller, significant predictor in their study was stress symptoms, alluding to the importance of the perceived need for the intervention.

This critique has been brought up repeatedly against models of technology acceptance. The use of a technology is not an end of its own, determined by how useful and user-friendly, but also by the perceived need for it, that is the task-technology fit [45,46]. According to Füllemann et al [47], an awareness for employee health is not yet present in many organizations, and hence, there may not have been a perceived need for an intervention to address this issue. The same is also implied in the Rogers [18] theory of innovation, which for the stage of attitude formation specifies that attributes not only inherent to but also external to the innovation, such as the need for it or its compatibility with other tools, are of relevance. The concept of fit is recognized in the intervention literature as well. Randall and Nielsen [48] proposed person-intervention fit and environment-intervention fit as 2 dimensions that acknowledge the complex social environment in which interventions occur and that provide a possible answer to why the same intervention sometimes succeeds and sometimes fails.

Given the participatory nature of wecoach, it makes sense that factors relating to the fit on the team level strongly contributed to its acceptance. CapTeam was the strongest contributor to acceptance in our regression analysis and reached significance in the absence of other predictors. The availability of resources on the organizational level and alignment with organization goals, as indicated by CapOrg, however, did not seem immediately relevant for the acceptance of wecoach, although it could be speculated that those, alongside the facilitating conditions, might gain salience in the actual implementation.

The 3 items of the CapTeam scale assessed competence, motivation, and identity, and a closer inspection of the items revealed that 2 of them (*Our team is motivated to do such team development* and *I think that such team development fits well with our team culture*) correlated highly with behavioral intention, while the third one (*Our team has the competences necessary to undertake such team development*) did not. It can be assumed then that the perceived motivation as well as fit with the team climate were the main drivers of intention to use wecoach. Note that these aspects relate entirely to the procedural aspect of wecoach, not the technological one. This could be particularly relevant in a highly collaborative work environment, such as nursing.

Consisting of not only a novel technological approach but also a novel approach to leadership and team development, wecoach may be too complex a tool to be suitably assessed with technology-related variables of UTAUT alone. Indeed, the intervention aspect of wecoach may have been more salient to the participants than the technology aspect of it. It would be interesting to further examine how users perceive and frame wecoach along these 2 dimensions.

As interventions become more sophisticated and more complex, especially in the work context, it is important to acknowledge the limitations of UTAUT and to recommend careful and deliberate selection of variables matched to the level at which innovations occur in order to better understand acceptance. Such fit-related aspects, informed by implementation science and intervention research, may serve as a gateway that determine acceptance before aspects such as usefulness or user-friendliness are even relevant. UTAUT may thus still be a suitable, although not a sufficient model, to understand the acceptance of complex technologies, and enhancing models with carefully selected variables can support researchers and practitioners in detecting the appropriate level to address facilitators of and barriers to their acceptance.

### Limitations and Outlook

Several limitations need to be considered in interpreting our findings. First, our sample of 32 was small and represented only a tiny fraction of the persons we invited to participate. This means that our findings are difficult to generalize to a broader population of nurse managers, despite the satisfactory post hoc power analysis. This also increases the likelihood that our sample was biased and already interested in or open to workplace health promotion or digital interventions. Furthermore, although substantial efforts were undertaken to include staff working in nursing homes, only 2 (6.25%) participants did, limiting the conclusions that can be drawn about that setting. As with any study attempting predictions, longitudinal data would have allowed us to strengthen causal assumptions between the assessed variables. The inclusion of moderators might have also enhanced the predictive power of our model. However, although they contributed substantial variance explanation in the original UTAUT publication study [26], their inclusion may no longer be the most feasible approach [40]. In addition, in our study, sample size limitations did not permit their inclusion. Being an exploratory study examining the acceptance of a complex online-based leadership and team development intervention, qualitative data could yield valuable additional insights into the drivers of the different predictors, especially an in-depth exploration of the perceived motivation on the team level and fit with team culture. It would also be interesting to further understand in what terms users framed wecoach—whether they perceived it more as technology or a team development method.

### Conclusion

Our study found that CapTeam is the only significant predictor of the intention to use wecoach. This implies that for successful dissemination of such a digitally supported participatory tool, the fit to the team, its culture, and its motivation are of much greater relevance than its technological aspects.

UTAUT has previously been 1 of the dominant models to determine acceptance of new technologies. Our findings suggest that in the case of complex technologies, this may not be the most appropriate model. As new technologies and digital interventions become more complex, it is important to supplement acceptance models through the careful selection of variables matched to the level at which the innovations occur. This can help researchers and practitioners identify the appropriate level to more fully understand acceptance and to address related barriers and facilitators to implementation and use of innovations.

## Abbreviations

CapOrg: capacities on the organizational level
CapSelf: capacities on the individual leader's level
CapTeam: capacities on the team level
OHD: organizational health development
UTAUT: unified theory of acceptance and use of technology
